# Pyroptosis inhibition improves the symptom of acute myocardial infarction

**DOI:** 10.1038/s41419-021-04143-3

**Published:** 2021-09-16

**Authors:** Wenju Liu, Junwei Shen, Yanfei Li, Jiawen Wu, Xiaoli Luo, Yuanyuan Yu, Yuhan Zhang, Liang Gu, Xiaobai Zhang, Cizhong Jiang, Jue Li

**Affiliations:** 1 Key Laboratory of Spine and Spinal Cord Injury Repair and Regeneration of Ministry of Education, Orthopaedic Department of Tongji Hospital, Shanghai Key Laboratory of Signalling and Disease Research, School of Life Sciences and Technology, Tongji University, 200092 Shanghai, China; 2 Shanghai Pudong New Area Mental Health Center, Tongji University School of Medicine, 200124 Shanghai, China; 3 Shanghai University of Medicine and Health Sciences Affiliated Zhoupu Hospital, Shanghai University of Medicine and Health Sciences, 201318 Shanghai, China; 4 Key Laboratory of Arrhythmias, Ministry of Education, China, Tongji University School of Medicine, 200124 Shanghai, China

**Keywords:** Transcriptomics, Cardiovascular diseases

## Abstract

Acute myocardial infarction (AMI), the leading cause of mortality worldwide, is a rapidly developing and irreversible disease. Therefore, proper prompt intervention at the early stage of AMI is crucial for its treatment. However, the molecular features in the early stage have not been clarified. Here, we constructed mouse AMI model and profiled transcriptomes and proteomes at the early stages of AMI progress. Immune system was extensively activated at 6-h AMI. Then, pyroptosis was activated at 24-h AMI. VX-765 treatment, a pyroptosis inhibitor, significantly reduced the infarct size and improved the function of cardiomyocytes. Besides, we identified that WIPI1, specifically expressed in heart, was significantly upregulated at 1 h after AMI. Moreover, *WIPI1* expression is significantly higher in the peripheral blood of patients with AMI than healthy control. WIPI1 can serve as a potential early diagnostic biomarker for AMI. It likely decelerates AMI progress by activating autophagy pathways. These findings shed new light on gene expression dynamics in AMI progress, and present a potential early diagnostic marker and a candidate drug for clinical pre-treatment to prolong the optimal cure time.

## Introduction

Acute myocardial infarction (AMI) and its sequelae are leading causes of mortality around the world, leading to more than 4 million deaths in the United States and European countries each year [1, 2]. Moreover, it is a rapidly developing and irreversible disease since the cardiomyocytes lost their ability to regeneration in adults [3]. Most of the AMI patients develop into heart failure and eventually die of it [1]. In addition to high mortality, the financial burden of AMI is tremendous. For instance, the disease cost the global economy an estimated US $863 billion worldwide in 2010 [4]. Fortunately, if the AMI patients could be diagnosed and treated timely and promptly, not only the therapeutic outcome will be improved dramatically, but also the costs will decrease sharply due to the reduction in hospitalization time. As such, diagnosis as soon as possible is the crucial factor to improve the AMI treatment. Cardiac troponins (cTnT and cTnI) are the gold clinical diagnostic biomarkers for AMI [5]. The high-sensitivity cardiac troponin (hs-Tn) assays are very sensitive but less specific for AMI [6–8]. Therefore, there is a dire need to pinpoint novel AMI biomarkers that are earlier than cTnT in order to improve patients’ clinical outcomes.

Owing to the rapid, irreversible decline of cardiomyocyte function, the optimal treatment window of AMI is very short [9]. As a result, prolonging the optional window through pre-treatment is extremely important. Nitroglycerin (GTN), first used to relieve angina in 1867, remains the first-line pre-treatment for AMI [10]. GTN achieves its benefit by giving rise to nitric oxide (NO), causing vasodilation and increasing blood flow to the myocardium [11]. Although GTN alleviates the symptom and improves clinical outcomes of AMI, it could not protect the cardiomyocytes [11]. Therefore, large numbers of AMI patients receiving GTN pre-treatment still deteriorate relentlessly and eventually die of heart failure. Searching for a feasible and effective pre-treatment for cardiomyocyte protection after AMI remains a central goal in cardiac biology.

Protein detection plays a crucial role in cardiovascular disease diagnosis. For instance, cardiac troponins, such as cTnT and cTnI, are important diagnostic indicators in AMI [12]. Searching for protein markers during AMI not only has good clinical values but also provides molecular insights into the underlying pathology. Mass spectrometry (MS) is an effective technology for proteome analysis with obvious limitations [13, 14]. For example, with this method, Peng et al. [15] revealed that ENH2, which belongs to the PDZ-LIM protein family, is highly expressed after AMI, whereas the differentially expressed proteins are rare. One advance in MS-based targeted proteomics is data-independent acquisition mass spectrometry (DIA-MS) [16, 17]. By characterizing different molecules produced from different samples, this method combined with improved bioinformatics analyses is more sensitive in detecting peptides or proteins that are usually missing in traditional MS experiments [18, 19]. The present study is designed to investigate the cellular and molecular changes in different AMI stages in a mouse model through the combination of transcriptome and proteome analyses.

## Materials and methods

### Animals

Thirty male C57BL/6 mice (8 weeks old) were purchased from Shanghai Slake Experimental Animal Co., Ltd. The mice were randomly divided into different experimental groups. They were anesthetized by intraperitoneal injection of sodium pentobarbital (50 mg/kg). AMI was performed by ligation of the proximal left anterior descending coronary artery. Subsequently, the mice were sacrificed, and the infarcted myocardium was obtained for further experiments. For the VX-765 treatment, the VX-765 (50 mg/kg) was dissolved in the mix (2% DMSO + 30% PEG300 + ddH_2_O). It was intraperitoneally injected into mice 20 min after MI.

### Echocardiography

The echocardiography was performed with a VisualSonics Vevo 2100 Imaging System (VisualSonics Inc, Toronto, Canada). Mice were anesthetized with 1% isoflurane (Sigma Aldrich, MO, USA) supplemented with oxygen via a vaporizer. M-mode measurements were used to detect LV dimensions, such as left ventricular internal dimension in diastole (LVID:d), left ventricular internal dimension in systole (LVID:s). The LV ejection fraction (EF%) was calculated as follows: (LVID:d – LVID:s) / LVID:d × 100.

### Masson’s trichrome staining

For masson’s trichrome staining, the AMI tissues were fixed with 4% paraformaldehyde (pH 8.0) overnight, then embedded in paraffin, and sectioned (6 μM). The infarction sections were stained with hematoxylin & eosin (H&E) and imaged under high (×400) magnification using alight microscope (Leica, Germany) to determine the extent of tissue damage. Heart sections were stained with Masson’s trichrome staining and visualized under low (×12.5) and high magnification. Infarct size was evaluated according to the ratio of infarcted tissue compared with total endocardial circumference.

### Quantitative reverse transcription-polymerase chain reaction (qRT-PCR)

Total RNA was extracted with the RNAiso Reagent (TaKaRa, Japan) in accordance with the manufacturer’s instructions. 500 ng of total RNA was reversely transcribed using the PrimeScript RT reagent kit (TaKaRa). The resulting cDNAs were detected by SsoAdvanced™ Universal SYBR® Green Supermix (Bio-Rad, Berkeley, USA). Primer sequences are listed in Supplementary Table 1. The fold changes were analyzed via the 2^−△△CT^ method.

### Western blots

The AMI tissues were lysed using strong RIPA buffer containing Halt Protease Inhibitor Cocktails (Thermo, Waltham, USA). Protein concentrations were evaluated with a bicinchoninic acid assay kit (Beyotime, Nantong, China). Primary antibodies targeting to beta actin (ab8226, Abcam, Cambridge, UK), CASPASE1 (ab138483, Abcam), WIPI1 (ab128901, Abcam) were incubated with targeted proteins at 4 °C overnight, followed by incubating with an appropriate horseradish peroxidase-conjugated secondary antibodies. Detection of horseradish peroxidase was performed with the Super Signal West Pico Chemiluminescent Substrate (Pierce).

### Preparation of RNA-seq library

Briefly, mRNA was purified from total RNA using poly-T oligo-attached magnetic beads. Fragmentation was carried out using divalent cations under elevated temperature in NEBNext First Strand Synthesis Reaction Buffer (5X). First strand cDNA was synthesized using random hexamer primer and M-MuLV Reverse Transcriptase (RNase H). Second-strand cDNA synthesis was subsequently performed using DNA Polymerase I and RNase H. Remaining overhangs were converted into blunt ends via exonuclease/polymerase activities. After adenylation of 3ʹ ends of DNA fragments, NEBNext Adaptors with hairpin loop structure were ligated to prepare for hybridization. In order to select cDNA fragments of preferentially 250−300 bp in length, the library fragments were purified with AMPure XP system (Beckman Coulter, Beverly, USA). Then 3 μl of USER Enzyme (NEB, USA) was used with size-selected, adaptor-ligated cDNA at 37 °C for 15 min followed by 5 min at 95 °C before PCR. Then PCR was performed with Phusion High-Fidelity DNA polymerase, Universal PCR primers and Index (X) Primer. At last, PCR products were purified (AMPure XP system) and library quality was assessed on the Agilent Bioanalyzer 2100 system. Finally, the library preparations were sequenced on an Illumina Novaseq platform and 150 bp paired-end reads were generated.

### RNA-seq data processing and analysis

Firstly, cutadapt (V1.18) [20] was used to remove adaptor sequences, low-quality bases and reads shorter than 50 bases with parameters “-a AGATCGGAAGAGC -A AGATCGGAAGAGC -trim-n -m 50 -q 20,20”. Next, the trimmed clean data were mapped to mm10 reference genome using Hisat2 (V2.1.0) [21] with parameters “-dta-cufflinks -no-discordant”. After that, gene expression levels were quantified as Fragments Per Kilobase per Million mapped reads (FPKM) by stringtie (V1.3.4d) [22]. Genes with FPKM < 1 in all samples were filtered, and FPKM values of replicates were averaged.

Gene expression comparisons between each AMI sample and the control sample were performed by cufflinks (V1.3.0) [23] with parameters “-multi-read-correct”. Then, the differentially expressed genes (DEGs) were identified with *P* value < 0.05 and fold change (FC) of FPKM larger than 2.

### Protein sample preparation

Nine myocardium samples were lysed with lysis buffer (1% SDS, 7 M urea, 1× Protease Inhibitor Cocktail (Roche Ltd. Basel, Switzerland)) and centrifuged at 15,000 rpm for 15 min at 4 °C. The supernatant was collected, and the protein concentration of the supernatant was determined using the BCA protein assay (Beyotime Ltd. Shanghai, China). The protein samples were adjusted to 100 μl with 8 M urea. Then, 2 μl of 0.5 M TCEP was added, and the samples were incubated at 37 °C for 1 h. Next, 4 μl of 1 M iodoacetamide was added to each sample, followed by incubation for 40 min in the dark at room temperature. After that, five volumes of −20 °C pre-chilled acetone was added to precipitate the proteins overnight at −20 °C. The precipitates were washed by 1 ml of pre-chilled 90% acetone aqueous solution twice and then re-dissolved in 100 μl of 100 mM TEAB. Sequence-grade modified trypsin (Promega, Madison, WI) was added at the ratio of 1:50 (enzyme:protein, weight:weight) to digest the proteins at 37 °C overnight. The peptide mixture was desalted by a C18 ZipTip, quantified by the PierceTM Quantitative Colorimetric Peptide Assay (23275) and then lyophilized by a SpeedVac.

### High pH reverse-phase separation

The lyophilized peptide mixture was re-dissolved in buffer A (20 mM ammonium formate in water, pH 10.0, adjusted with ammonium hydroxide), then fractionated by high-pH separation using an Ultimate 3000 system (Thermo Fisher Scientific, MA, USA) connected to a reverse-phase column (XBridge C18 column, 4.6 × 250 mm, 5 μm (Waters Corporation, MA, USA)). High-pH separation was performed using a linear gradient, starting from 5% B to 45% B in 40 min (B: 20 mM ammonium formate in 80% ACN, pH 10.0, adjusted with ammonium hydroxide). The column was re-equilibrated at the initial condition for 15 min. The column flow rate was maintained at 1 ml/min, and the column temperature was maintained at 30 °C. Ten fractions were collected; each fraction was dried in a vacuum concentrator for the next step.

### DIA:nano-HPLC-MS/MS analysis

The peptides were re-dissolved in 30 μl of solvent A (0.1% formic acid in water) and analyzed by online nanospray LC-MS/MS on an Orbitrap Fusion Lumos (Thermo Fisher Scientific, Bremen, Germany) coupled to a Nano ACQUITY UPLC system (Waters Corporation, Milford, MA). Ten microliters of peptide was loaded onto the trap column (Thermo Fisher Scientific) with a flow rate of 300 nl/min and subsequently separated on the analytical column (Acclaim PepMap C18, 75 μm × 15 cm) with a nonlinearly increased gradient from 3% B (0.1% formic acid in ACN) to 7% B in the first 3 min, from 7% B to 20% B in the next 3−83 min, from 20% B to 32% B in the next 83−107 min, and from 32% B to 90% B in the next 107−108 min, followed by holding at 90% B for 12 min. The column temperature was 40 °C. An electrospray voltage of 2.1 kV versus the inlet of the mass spectrometer was used. The mass spectrometer was run under data-independent acquisition mode and automatically switched between MS and MS/MS mode. The parameters were: (1) MS: scan range (m/z) = 350−1200; resolution = 120,000; AGC target = 400,000; maximum injection time = 50 ms; (2) HCD-MS/MS: isolation window = 3 Th, resolution = 30,000; AGC target = 1,000,00; maximum injection time = 72 ms; collision energy = 35%; stepped CE = 5%.

### DIA-MS data processing and analysis

The raw data of DIA-MS were processed and analyzed by Spectronaut Pulsar X with default settings (BGS Factory Settings) to generate an initial target list, which contained 48,880 precursors, 37,512 unique peptides, 7381 proteins and 4916 protein group. Spectronaut was set up to search the database of Uniprot-proteome-mus-musculus_201711 (51,946 entries) assuming trypsin as the digestion enzyme. Carbamidomethyl (C) was specified as the fixed modification. Oxidation (M) was specified as the variable modifications. Qvalue (FDR) cutoff on precursor and protein level used 1%. All selected fragment ions passing the filters were used for quantification.

To quantify the protein abundance levels across samples, we summed up the most abundant peptides for each proteins according to the previous method [16, 24, 25]. Then, the protein expression values of replicates were averaged and the data matrix was log2 transformed for later statistical and bioinformatics analysis.

The differentially expressed proteins (DEPs) between samples of every time point after AMI and the control sample were identified with *P* value < 0.05 (Student’s *t* test) and absolute log2 ratio > 0.58.

### Functional annotations and enrichment analysis

To annotate the enriched functions of selected genes or proteins which were presented unique expression patterns, we performed Gene Ontology (GO) enrichment analyses of biological processes in DAVID Bioinformatics Resources (v6.8) with default settings. The Gene Set Enrichment Analysis (GSEA, V3.0) was performed, in which the list of genes were ranked by the expression fold change of the AMI-sample/Control-sample, and then a logistic model is used to detect gene sets (specialized gene sets of GO biological processes downloaded from MSigDB (https://www.gsea-msigdb.org/gsea/index.jsp) and Gene Ontology Resource (http://geneontology.org/)) that are consistently associated to high or low values in the ranked lists. The *P* value < 0.05 from the logistic regression was used to determine the statistical significance of GSEA analysis.

### Immune cell type abundance

To characterize the changes of immune cell composition after myocardial infarction, we used CIBERSORT [26] with specialized signature matrix. In detail, the signature matrix contains 644 cell-type-specific marker genes, which can distinguish 27 cell types including myocardial cell, cardiac fibroblasts and 25 immune cell types. The marker genes of 25 immune cell types were selected according to the previous method [27]. The data of myocardial cells and cardiac fibroblasts were downloaded from GSE89885, and the marker genes were defined as those specifically highly expressed in one cell type (FPKM > 10) but not expressed in other cell types (FPKM < 0.5).

### Construction of protein regulatory network related to pyroptosis

To reveal the regulatory relationship between genes and proteins, we used STRING (V11.0, https://string-db.org/) to predict the protein−protein interaction. Firstly, we generated the proteins list that were involved in pyroptosis pathway and specifically expressed in AMI samples. Next, the protein list was submitted to the STRING and the predicted results were selected with their combined score >0.4. Finally, the gene−protein regulatory network related to pyroptosis was graphically presented by Cytoscape (V3.7.0) [28], of which genes and proteins were distinguished by the shape of network nodes.

### Visualization of transcriptome and proteome data

R Studio (https://rstudio.com/) was used to run custom R scripts to perform Principal component analysis (PCA) and correlation analysis. The graphs were generally constructed by ggplot2 packages, such as bar graphs, volcano plots, and line plots.

### Electron microscopic study

Autologous emboli were analyzed by scanning electron microscope (SEM). After 3-h automatic retraction, specimens were washed three times with phosphate buffer, fixed for 120 min in 2% glutaraldehyde and rinsed three times with phosphate buffer. Samples were then fixed for 120 min with osmic acid, rinsed, and dehydrated in a graded series of ethanol concentrations (50, 70, 90 and 100%) over a period of 40 min and further dehydrated in a graded series of concentrations (50, 70, 90, 100%) of isoamyl acetate-ethanol solvent. The clots were dried with hexamethyldisilazane for 10 min and fractured naturally through pulling to obtain a fracture surface for analysis. Finally, clots were coated with gold-palladium prior to examination in an SEM (TM-1000, HITACHI).

### Statement of ethics

All animal experimental protocols adhered to the National Institutes of Health *Guide for the Care and Use of Laboratory Animals* (NIH Publications No. 8023, revised 1978) and protocols approved by the Institutional Animal Care and Use Committee of Tongji University (Shanghai, China).

### Statistical analysis

All the results represented three or more independent experiments with the data expressed as the mean ± SD. Differences between the control and treatment groups were analyzed using Student’s *t* test in Graphpad.Prism 8. A *P* value < 0.05 was considered significant. The number of samples was declared in the corresponding figure legend section.

## Results

### Transcriptome and proteome profiling of mouse AMI model

**Fig. 1:**
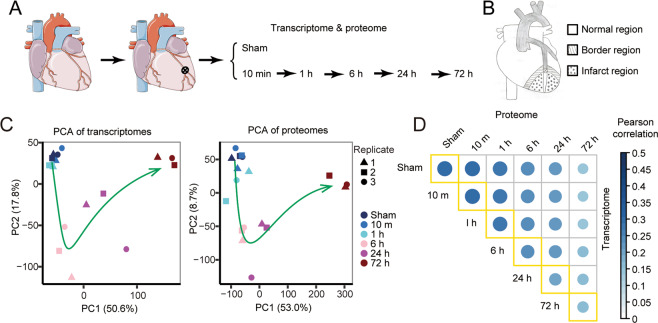
Transcriptome and proteome profiling of AMI model. **A** Schematic diagram of AMI model and the stages for transcriptome and proteome profiling. Eighteen mice were randomly divided into six groups. AMI was established by ligation of the proximal left anterior descending coronary artery indicated by the circled “x”. **B** Schematic diagram shows the infarct region indicated by dotted area. **C** Principal component analysis (PCA) of the transcriptome (left) and the proteome (right). Arrowed lines indicate the trajectory of AMI progress. **D** The correlation between the transcriptome and proteome profiles.

In order to characterize the dynamic molecular changes and screen the molecular biomarkers in the early stages of AMI in vivo, we generated mouse AMI model by ligation of the proximal left anterior descending coronary artery. The infarct left ventricle tissues isolated at six time points spanning 0 min to 72 h were acquired for RNA-seq and DIA-MS (Fig. 1A). The infarct regions are mainly in the ventricular apical regions (Fig. 1B). Cardiac ultrasound results showed that the ejection fraction (EF) value in the AMI model group was significantly lower than that in the control group (Supplementary Fig. 1A). These results indicate that the mouse model effectively mimics the AMI process. Subsequently, we examined the expression patterns of the known cell-type-specific markers for cardiac myocytes (CM) and cardiac fibroblasts (CFs). As expected, transcriptome data showed CM marker genes (*Slc2a4*, *Cs*, *Atp2a2*, *Tnnt2*, *Pdk2*) highly expressed in the sham and AMI samples, while CF maker genes (*Ltbp2*, *Comp*, *Cilp*) kept low expression until 72 h after AMI (Supplementary Fig. 1B). Proteome data also showed CM marker proteins (SLC2a4, TNNT2, ATP2A2, CS) in high abundance levels (Supplementary Fig. 1C).

The principal analysis (PCA) of the transcriptome profiles showed AMI samples in the extremely early stage (before 1 h) clustered together with sham samples, while samples of the later three time points separated following the trajectory to the development of AMI (Fig. 1C). These results suggested 6 h is the key time point of AMI process. Consistently, the PCA result of the proteome data also showed 6 h was the significant turning point (Fig. 1C). Interestingly, we found 1-h AMI sample obviously separated from sham samples in proteome (Fig. 1C). This indicated the involvement of post-transcriptional regulation. Correlation analysis between mRNA abundance and total protein levels in AMI samples revealed moderate correlations and these correlations weakened as AMI progressed (Fig. 1D). This further indicated the post-transcriptional regulation in AMI process. These results imply the dynamic changes in the transcription and expression of genes in AMI process.

### Myocardial tissue entered the immune-activated state at the early stage of AMI

**Fig. 2:**
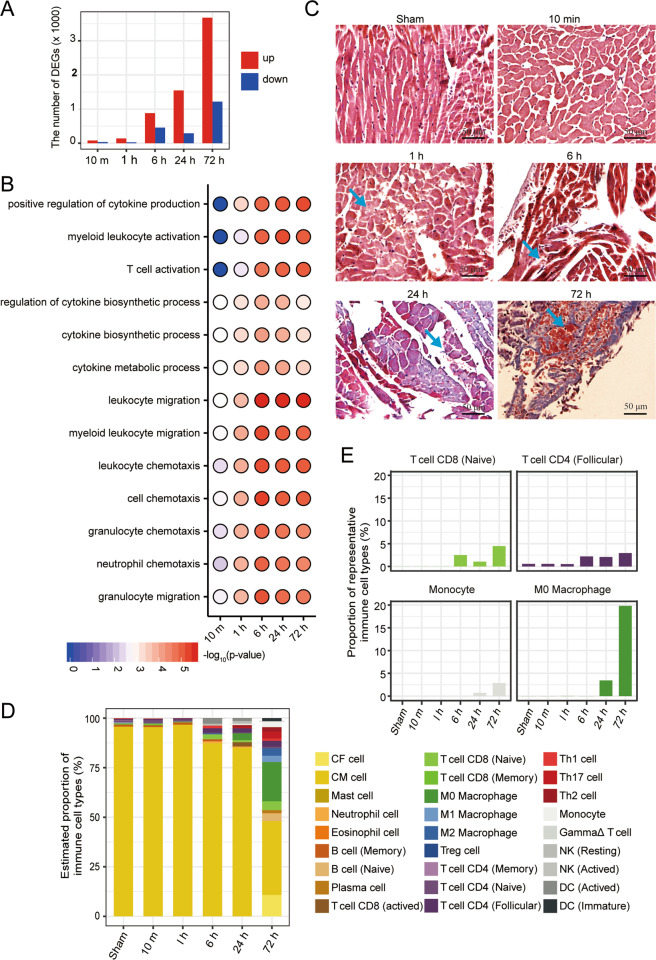
Immune response at the early stage of AMI. **A** The number of the differentially expressed genes (DEGs) between AMI samples and the control sample. Red: upregulated, blue: downregulated. **B** Bubble diagram shows the significantly enriched GO terms (biological processes) of upregulated DEGs. **C** Masson staining results show the morphologic changes in AMI samples compared to the control sample. The arrows indicate the damaged cardiomyocytes at 1, 6 and 24 h. At 1 h, the cytoplasm of the cardiomyocytes begins to swell, and then the cells begin to disintegrate at 6 h; the fibrosis were found around the cardiomyocytes at 24 h, and the space made by the dead cardiomyocytes was occupied by red blood cells and immune cells at 72 h (Bar = 50 μm). **D** The composition of immune cells in the infarcted myocardia at different stages of AMI. CM cardiac myocytes, CF cardiac fibroblasts. **E** The dynamic proportion of the representative types of immune cells in the course of AMI development, indicating the activation of immune infiltration.

To explore the dynamic molecular changes in the early stage of AMI, we identified the DEGs and the DEPs between the AMI samples and the sham samples. Interestingly, the number of upregulated DEGs sharply increased in 6-, 24- and 72-h AMI samples (Fig. 2A). So were DEPs (Supplementary Fig. 2A). This further confirms that AMI 6 h is a critical stage when extensive molecular changes has occurred to facilitate AMI progress.

We further explored what functional events occurred in the progresses of AMI. GO analysis of the upregulated DEGs showed that the immunity-related pathways, such as myeloid leukocyte activation and cytokine biosynthetic process, were significantly activated (Fig. 2B). Similarly, the upregulated DEPs were also enriched for the immunity-related pathways, such as humoral immune response, leukocyte mediated immunity, etc. (Supplementary Fig. 2B). Consistently, the immunity-related genes were highly expressed at 6-h AMI and later stages (Supplementary Fig. 2C). These results suggested that the immune system was activated in the early stage of AMI.

To confirm the activated immune system, we detected the morphological changes of the AMI tissues. Masson staining results displayed that the cardiomyocytes decreased gradually, and the granulocytes continuously infiltrated into the infarction region (Fig. 2C). Thus, these results provided further evidence that myocardial tissue entered the immune-activated state at the early stage of AMI.

Next, we employed CIBERSORT, which is an approach to define the compositions of immune cell populations in tissues based on the expression profiles [26], to identify what kind of immune cells were involved in the inflammatory response in the early stage of AMI. The results showed that cardiac myocytes accounted for the majority of cells as expected, whose proportion decreased as AMI advanced. Contrarily, the proportion of immune cells gradually increased since AMI 6 h and had a precipitous augmentation at 72 h. Of note, the majority of immune cells is M0 macrophages (Fig. 2D). Specifically, the proportion of T-cell CD8 (naïve) and T-cell CD8 (follicular) increased from AMI 6 h while monocytes and macrophages showed an increased proportion since AMI 24 h (Fig. 2E). Previous studies have reported macrophages were critically involved in wound healing following myocardial infraction [29, 30]. Hence, T-cell CD8 (naïve) and T-cell CD8 (follicular) could play an important role in the inflammatory response at the early stage of AMI, which could be studied to attenuate the symptoms of AMI and improve heart function.

### Pyroptosis is activated in AMI

Interestingly, gene set enrichment analysis (GSEA) results revealed that the upregulated DEGs of AMI 72-h sample compared to the sham sample were significantly enriched for apoptosis (Supplementary Fig. 2D). Consistently, the apoptosis-related genes were highly expressed as AMI advanced (Supplementary Fig. 2E).

**Fig. 3:**
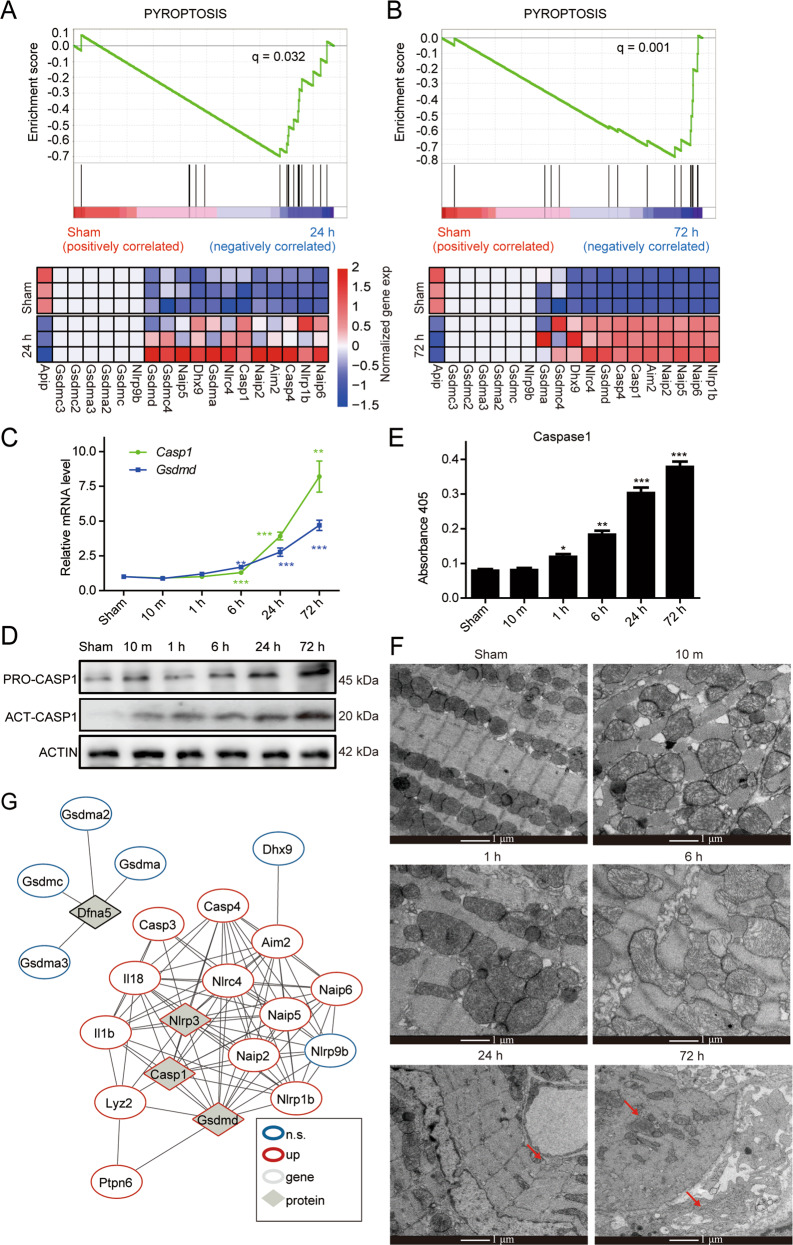
The pyroptosis signaling pathway is activated with the AMI development. **A**, **B** GSEA results: pyroptosis-related genes are upregulated in the infarcted myocardia at 24 h (**A**) and 72 h (**B**) of AMI model. **C** qRT-PCR results show the continuously increased expression levels of the representative pyroptosis marker genes along the AMI development (*n* = 3, **P < 0.01, ***P < 0.001, Student’s *t* test). **D** Western blotting shows the continuously increased protein levels of PRO-CASP1 and ACT-CASP1 along the AMI development. **E** The continuously increased activity of the pyroptosis marker gene *Caspase1* during the AMI development (*n* = 3, *P < 0.05, **P < 0.01, ***P < 0.001, Student’s *t* test). **F** Electronic microscopic results show the morphologic changes in the AMI samples compared to the sham samples. Arrows indicate pyroptotic bodies (Bar = 1 μm). **G** The network composed of the pyroptosis signaling pathway genes indicates the active interactions. n.s. not significant.

Previous studies have demonstrated that pyroptosis, a programmed cell death, is closely associated with cell apoptosis and is accompanied by inflammatory response [31]. Therefore, we evaluated whether pyroptosis plays an important role in AMI. We performed GSEA with the specified pyroptosis gene set, which was downloaded from Gene Ontology Resource and contained 20 genes. GSEA results revealed that the upregulated DEGs of both 24 and 72 h AMI 24-h samples compared to the sham samples were significantly enriched for pyroptosis. Most pyroptosis genes were highly expressed in the AMI samples (Fig. 3A, B). The core genes of the pyroptosis pathway *Gsdmd* and *Casp1* were significantly upregulated since AMI 1 h (Fig. 3C). The protein level of CASPASE1, a protein on which the pyroptosis pathway depends, also significantly increased in the AMI progress (Fig. 3D), so was its activity (Fig. 3E). Additionally, the electron microscopy confirmed the cellular morphology of pyroptosis in the AMI samples (Fig. 3F). All these results suggested the important role of pyroptosis in AMI.

To further reveal the mutual regulation of pyroptosis-related genes, protein−protein interaction network analysis was performed. The result showed apoptosis-depended proteins CASPASE1 and GSDMED, and NLRP3 (NACHT, LRR and PYD domains-containing protein 3) were at the core position of network (Fig. 3G). Other NLR family members (such as, Nlrp1b, Naip6, Naip2) and interleukin (such as, Il1b, Il18) tightly interacted in the network. Additionally, the expression patterns of genes in the regulation network were consistent (Supplementary Fig. 3). These findings provided guidance for researching the regulatory mechanism of pyroptosis during AMI.

### Pyroptosis inhibitor is a promising pre-treatment for AMI

**Fig. 4:**
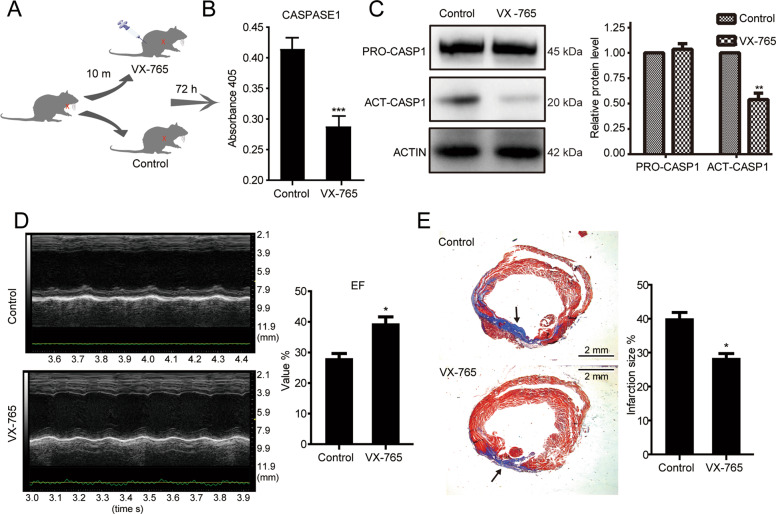
VX-765 treatment significantly reduces the infarction region of AMI. **A** Schematic diagram of the VX-765 treatment of AMI model. **B** Caspase1 activity is significantly decreased at 72 h AMI samples with VX-765 treatment (*n* = 3, ***P < 0.001, Student’s *t* test). **C** VX-765 treatment decreases the protein level of ACT-CASP1 not PRO-CASP1. Left: Western blotting analysis of pro-CASPASE1 and active-CASPASE1. Right: Quantification of pro-CASPASE1 and active-CASPASE1 of the Western blotting results (*n* = 3, **P < 0.01, Student’s *t* test). **D** Echocardiography results show the rescue effect of VX-765 treatment on AMI (*n* = 3, *P < 0.05, Student’s *t* test). **E** VX-765 treatment decreases the infarction region. Left: Masson staining results show the reduced size of the infarction region with VX-765 treatment. Arrows indicate the infarction region (blue). Right: quantification of the infarction region indicates VX-765 treatment significantly reduces the infarction region (*n* = 3, *P < 0.05, Student’s *t* test).

Since pyroptosis plays a pivotal role in AMI, we investigated whether pre-treatment with VX-765 (a specific CASPASE1 suppressor and pyroptosis inhibitor) could alleviate the symptom of AMI [32]. Indeed, we treated the AMI mice with VX-765 at the extremely early stage (10 min) of AMI (Fig. 4A) and found that the activity of CASPASE1 significantly decreased (Fig. 4B). The protein level of active CASPASE1 greatly decreased as well (Fig. 4C). Consequently, the EF value of the mice treated with VX-765 was profoundly improved 72 h after AMI (Fig. 4D). We further examined whether VX-765 can provide a long-term preservation for AMI and found that the infarct sizes in the VX-765 group were much smaller than that in the control group (Fig. 4E). Collectively, VX-765 treatment in the early stage can attenuate pyroptosis and improve the symptom of AMI by reducing the infarct region in a long period.

### *Wipi1* is a potential diagnostic biomarker for AMI

Biomarkers of cardiac injury have been used to diagnose AMI for over half a century but have the specificity limitation. Ideal biomarkers should show high sensitivity as early as possible after the onset of symptoms. Notably, AMI 1-h samples obviously separated from sham samples in the PCA results of proteome (Fig. 1D). This implied prominent changes in proteome as early as 1 h after AMI. It is also a hint that it is possible to screen protein markers from DEPs of 10 min and 1 h samples.

**Fig. 5:**
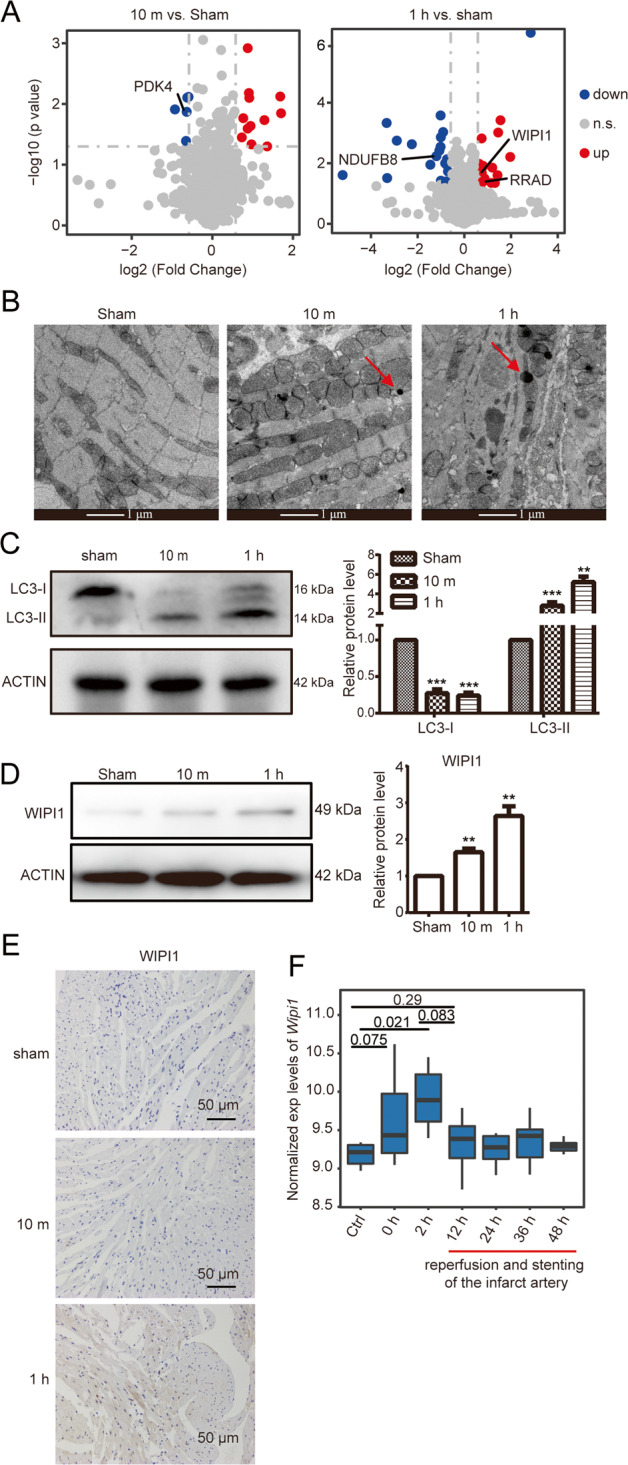
WIPI1 serves as a potential early biomarker for AMI. **A** Volcano plots show the differentially expressed proteins (DEPs) in the 10-min (left) and 1-h (right) AMI samples compared to the sham samples. Red dots: the upregulated proteins; Blue: the downregulated proteins; Gray: the not significantly changed proteins. **B** Electronic microscopic results show the morphologic changes in the 10-min and 1-h AMI samples compared to the sham samples. Arrows indicate autophagosomes (Bar = 1 μm). **C** Significantly increased expression levels of the autophagosome-marker gene LC3-II at the early stages of AMI. Left: Western blotting analysis of LC3-I and LC3-II in the 10-min and 1-h AMI samples. Right: quantification of the Western blotting signals of LC3-I and LC3-II (*n* = 3, **P < 0.01, ***P < 0.001, Student’s *t* test). **D** Left: Western blotting analysis of WIPI1 in the 10-min and 1-h AMI samples. Right: quantification of the Western blotting signals of WIPI1 (*n* = 3, **P < 0.01, Student’s *t* test). **E** Masson staining results show WIPI1 was significantly expressed in the tissue of the 1-h AMI samples. **F** Boxplot shows the expression levels of WIPI1 in peripheral blood from patients with AMI. Ctrl healthy person (Student’s *t* test).

There were 12 upregulated and five downregulated DEPs in the 10-min samples and 22 upregulated and 19 downregulated DEPs in the 1-h samples compared to the sham samples (Fig. 5A). Intriguingly, the upregulated DEGs (*Wipi1*, *Rrad*) and the downregulated DEGs (*Pdk4*, *Ndufb8*) were specifically highly expressed in adult heart (Supplementary Figs. 4 and 5A). This suggested that they were potential diagnostic markers for AMI. Further GO analysis revealed that the upregulated DEPs in the AMI 1-h samples compared to the sham samples were significantly enriched for autophagy GO terms (Supplementary Fig. 5B). Electron microscopy confirmed autophagic vesicles in the AMI 1-h sample (Fig. 5B). Besides, the protein level of LC3-II, a marker protein for autophagosome, was significantly increased (Fig. 5C). These results demonstrated that autophagy took place in the early stages of AMI, which may serve as a self-protection-mechanism of myocardial tissue. Coincidently, WIPI1, an important protein in autophagy pathway, was specifically highly expressed in the adult heart and was an upregulated DEP in the AMI 1-h group compared to the sham group (Fig. 5A, D and Supplementary Fig. 4). Immunohistochemical results also showed that AMI 1-h mice has elevated WIPI1 expression in myocardium tissue than the sham mice (Fig. 5E). Therefore, protein WIPI1 can serve as a potential early diagnostic biomarker for AMI.

To examine the efficacy of protein WIPI1 in clinic, we examined the expression of *Wipi1* in the peripheral blood cells of patients with early-stage AMI. The results showed that its expression level was significantly increased in AMI patients who had not been treated (0 and 2 h) compared to the control group. After successful mechanical reperfusion and stenting of the infarct artery (at MI 2 h), its expression significantly reduced and maintained at low expression level, which was slightly higher than that of the control group (Fig. 5F). These results further suggested that WIPI1 is a potential early diagnostic biomarker for AMI.

## Discussion

AMI is one of the most severe threats to human health. The vast majority of patients with AMI will develop into heart failure or eventually die [33, 34]. Therefore, we speculated that there should exist an important stage in AMI progress after which it becomes extremely difficult for cardiomyocytes to self-repair. We observed remarkable changes in both RNAs and proteins at 6 h of AMI (Fig. 2A and Supplementary Fig. 2A). Coincidently, 6 h is a vital time point for AMI clinical treatment. In contrast, there are only slight difference in RNAs and proteins at 1 h of AMI (Fig. 2A and Supplementary Fig. 2A). Therefore, 6 h after AMI is likely the such critical earliest stage for AMI progress. However, if we sampled between 1 and 6 h, remarkable changes in gene expressions could occur before 6 h.

Although treatment for AMI patients less than 6 h from onset has good clinical outcomes, a large number of patients miss the optimal cure time. Therefore, prolonging the optimal time is crucial to improve the clinical outcome. We found that pyroptosis was actively involved in AMI progress (Fig. 3) and pre-treatment with pyroptosis inhibitor VX-765 not only remarkably reduced the area of heart damage, but also improved the function of mouse hearts dramatically (Fig. 4). This result indicates that taking VX-765 before clinical treatment could extend the optimal time. Similarly, it is reported that the combined use of VX-765 and Ticagrelor, a P2Y12 antagonist, could decelerate the symptom of AMI and sustained infarct size reduction in rats [35]. NGT causes vasodilation and improves the blood flow to infarction size [36, 37], while VX-765 directly protects the cardiomyocytes. Thus, NGT and VX-765 are complementary in AMI therapy. Notably, a clinical II trial of VX-765 on epilepsy had already been taken [38]. Although the final therapeutic results are not good, it may be beneficial to its application on AMI since the clinical trial on epilepsy provides a large amount of clinical data, such as the safety data. Since increasing evidence demonstrates that VX-765 is beneficial to AMI, it is of great value to test its application value through large-scale clinical trial.

In addition to prolonging the optimal time of AMI treatment, diagnosis as early as possible is also a critical strategy to improve the treatment outcomes of AMI. Therefore, early diagnosis biomarkers for AMI are of great clinical value. We found that WIPI1 is such a diagnosis biomarker for AMI, whose expression is significantly upregulated in the very early stage of AMI (1 h) (Fig. 5A, D). Of note, although the increase of *Wipi1* mRNA in AMI serum is moderate (Fig. 5F), the protein level of WIPI1 in the AMI 1 h group is more than 1.5 times that in the sham group (Fig. 5D and Supplementary Fig. 4A). Therefore, WIPI1 can be a potential early diagnostic biomarker for AMI especially at protein level. In combination with WIPI1, the conventional clinical diagnostic markers cTnT and cTnI have the potential to improve the specificity of AMI diagnosis. Notably, the symptoms of some AMI patients are not significant, and they cannot feel pain until it is very serious [39, 40]. Therefore, daily real-time detection is of great importance for people at high risk of AMI. Currently, there are some electronic devices for detecting heart rate, such as electronic bracelets and sports watches, which can monitor the condition of the heart in real-time mode [41, 42], and related medical electronic products may play an important role in the prevention and treatment of AMI.

In conclusion, we detected changes in transcriptomes and proteomes of myocardial tissue at different time points of AMI, and found that 6 h after AMI was the earliest key time point when significant changes took place. Myocardial tissue entered the immune-activated state at the early stage of AMI. Pyroptosis was also activated in AMI. Pre-treatment of pyroptosis inhibitor VX-765 in the early stage greatly reduced the infarction size of AMI and improved the contractile function of the heart. Thus, VX-765 could be a potential target drug for AMI. Besides, autophagy occurred in the very early stage of AMI, which may be a form of self-protection of the heart. Protein WIPI1 specifically expressed in heart, a member of the autophagy pathway, was significantly increased within an hour after AMI. It could serve as a potential early diagnostic biomarker for AMI. Independent validation via clinical trials is necessary to determine whether the findings can translate to application.

## Supplementary information

aj-checklist

cddis-author-contribution-form

Suppl.Figures

## Data Availability

The raw sequence data reported in this paper have been deposited into the Gene Expression Omnibus (GEO) under GEO accession: GSE153494. The DIA raw data and the results reported at protein level as well as peptide level are available at iPROX: IPX0002295000, and the DIA data can also be freely downloaded from ProteomeXchange (http://proteomecentral.proteomexchange.org) Consortium via identifier PXD020193.

## Code availability

Code available on request from the corresponding authors.
