# Supplementary material for: Pyroptosis inhibition improves the symptom of acute myocardial infarction
Source: Cell Death Dis. 2021 Sep 16;12(10):852. doi: 10.1038/s41419-021-04143-3 (PMC8445977; doi:10.1038/s41419-021-04143-3)
Supplement: Supplementary file 2 — cddis-author-contribution-form [file 41419_2021_4143_MOESM2_ESM.pdf]

DECLARATION OF CONTRIBUTIONS TO ARTICLE

ADMC

Manuscript Number: CDDIS-21-1929R

Journal Name: *Cell Death & Disease* (the 'Journal')

Proposed Title of the Contribution:

Pyroptosis inhibition improves the symptom of acute myocardial infarction (the 'Contribution)

Author(s):

Wenju Liu, Junwei Shen, Yanfei Li, Jiawen Wu, Xiaoli Luo, Yuanyuan Yu, Yuhan Zhang, Liang Gu, Xiaobai Zhang, Cizhong Jiang, Jue Li (the 'Authors')

For all *CDDis* articles, each person named as an author in the published version must be able to show he or she has contributed substantially to the article.

Authorship credit should be based on 1) substantial contributions to conception and design, acquisition of data, or analysis and interpretation of data; 2) drafting the article or revising it critically for important intellectual content; and 3) final approval of the version to be published. Authors should meet conditions 1, 2 and 3.

Any person who cannot be shown to have made a substantial contribution to the article cannot be listed as an author in the final version. The name of any person who is deemed to have made a minor contribution can, however, appear in the Acknowledgments section of the article.

Please complete the table below to indicate the contributions of all named authors to the manuscript.

Author Full Name:	Specification of Contribution to the Manuscript:
Wenju Liu	did most data analyses and wrote the manuscript
Junwei Shen	did most experiments and wrote the manuscript
Yanfei Li	provided most samples
Jiawen Wu	helped with experiments
Xiaoli Luo	helped with experiments
Yuanyuan Yu	helped with data analyses
Yuhan Zhang	helped with data analyses
Liang Gu	helped with data analyses
Xiaobai Zhang	helped with data analyses
Cizhong Jiang	conceived and designed the experiments and wrote the manuscript
Jue Li	conceived and designed the experiments

Please complete the table below to indicate the contributions of all named authors to the figures.

Figure 1:

A: Junwei Shen, Yanfei Li
B: Junwei Shen
C: Wenju Liu
D: Wenju Liu

Figure 2:

A: Wenju Liu, Yuanyuan Yu
B: Wenju Liu
C: Junwei Shen, Jiawen Wu
D: Wenju Liu
E: Wenju Liu

Figure 3:

A: Wenju Liu
B: Wenju Liu
C: Junwei Shen
D: Junwei Shen
E: Junwei Shen
F: Junwei Shen, Yanfei Li
G: Wenju Liu, Yuhan Zhang

Figure 4:

A: Yanfei Li
B: Yanfei Li
C: Junwei Shen
D: Yanfei Li
E: Yanfei Li, Xiaoli Luo

Figure 5:

A: Wenju Liu
B: Junwei Shen, Yanfei Li
C: Junwei Shen
D: Junwei Shen
E: Junwei Shen
F: Wenju Liu, Liang Gu, Xiaobai Zhang

Figure 6:

Signed for and on behalf of the Author(s):	Print Name:	Date:
[illegible]	Cizhong Jiang	2021/7/12
