# Supplementary material for: Pyroptosis inhibition improves the symptom of acute myocardial infarction
Source: Cell Death Dis. 2021 Sep 16;12(10):852. doi: 10.1038/s41419-021-04143-3 (PMC8445977; doi:10.1038/s41419-021-04143-3)
Supplement: Supplementary file 3 — Suppl.Figures [file 41419_2021_4143_MOESM3_ESM.pdf]

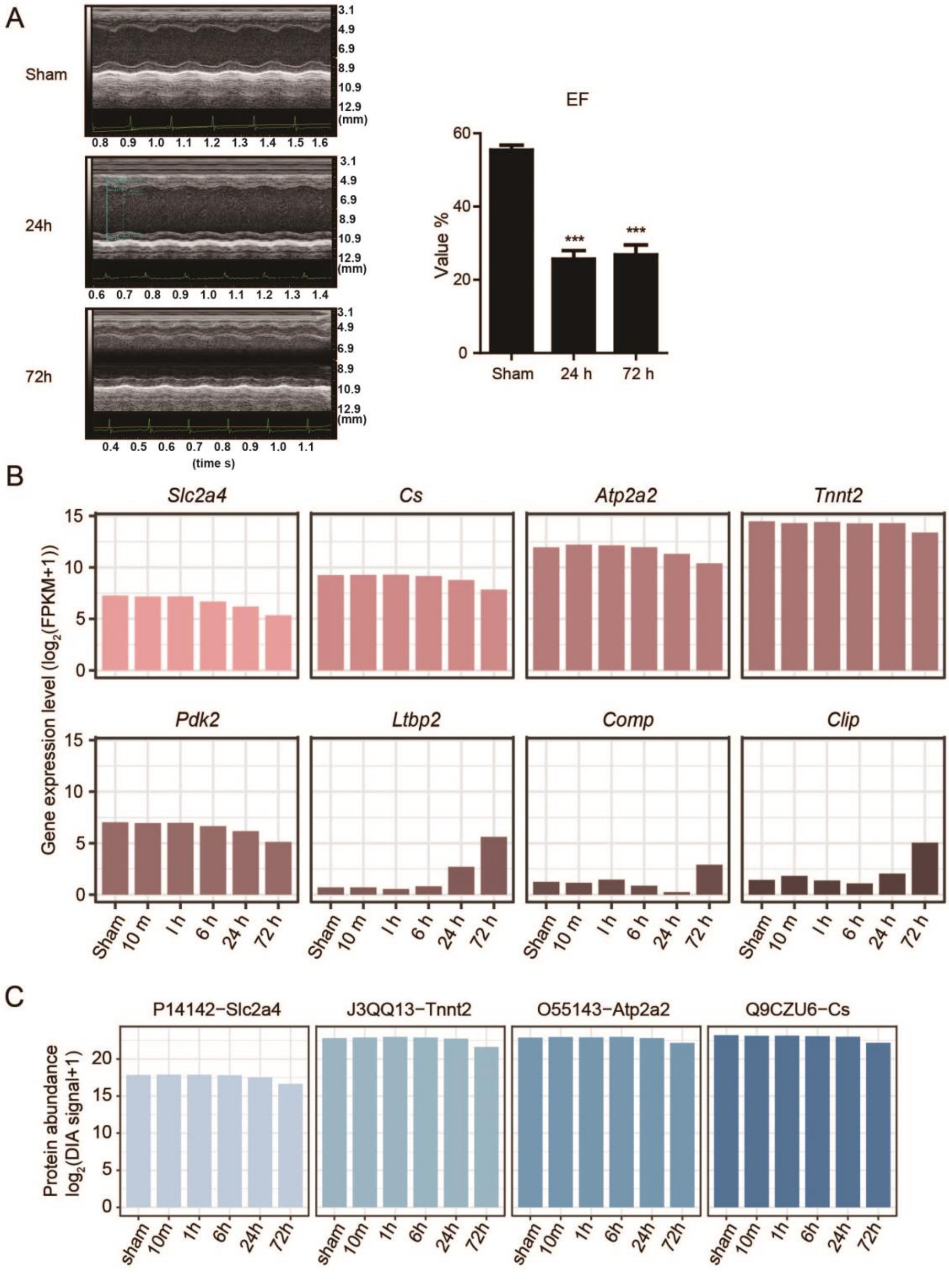

Supplementary Figure 1 Echocardiography and the expression patterns of the representative markers in AMI samples.

A Echocardiography results show the decline of the contractile function of the heart in AMI samples. (***: $p < 0.001$, Student's t-test)

B Bar plots show the expression patterns of cardiomyocyte and fibroblast marker genes.

C Bar plots show the protein abundance level of cardiomyocyte marker genes.

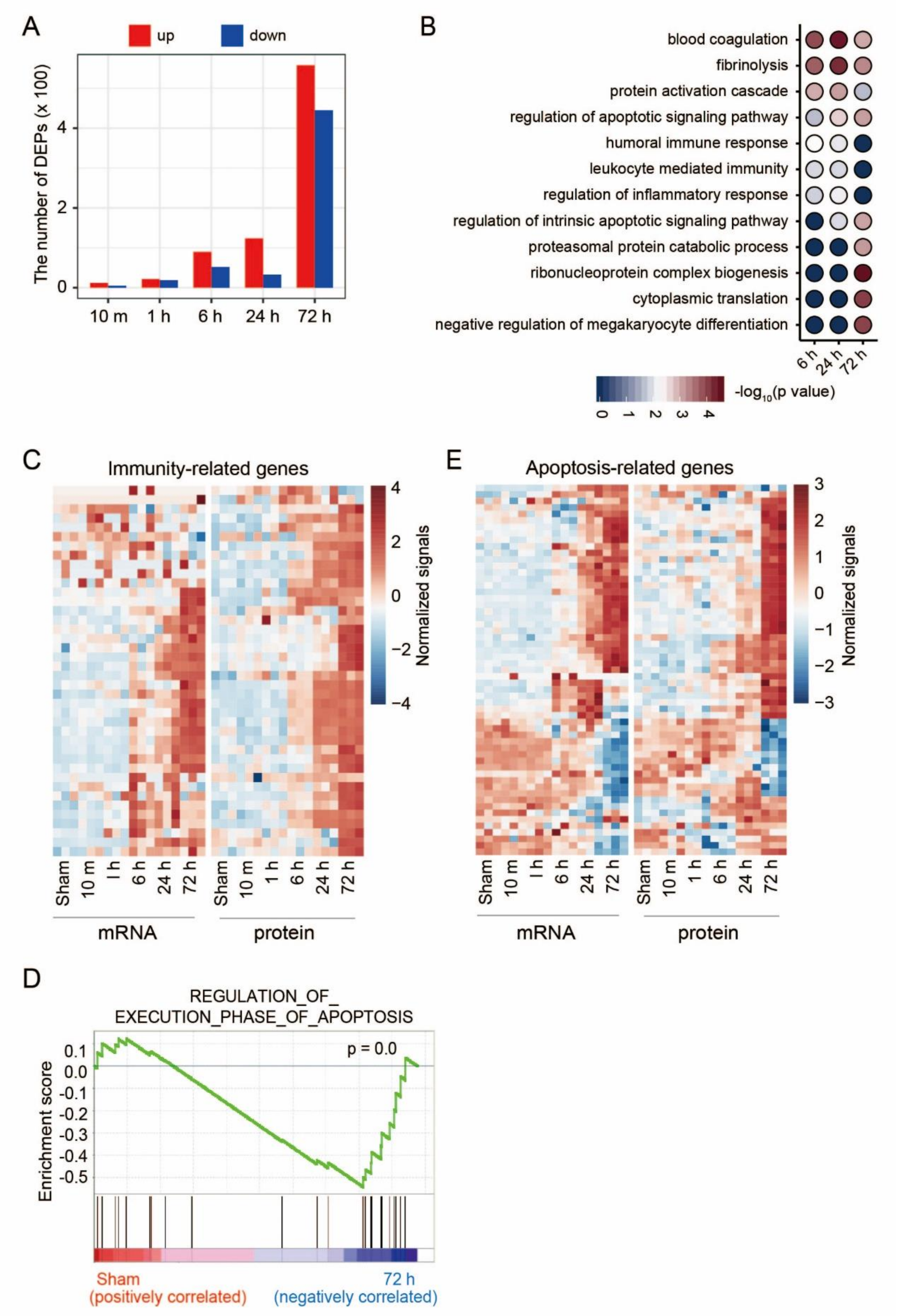

Supplementary Figure 2 Immune response and apoptosis are activated during the AMI

development.

A The number of the differentially expressed proteins (DEPs) in the AMI samples compared to the sham samples.

B Bubble diagram shows the significantly enriched GO terms (biological processes) of up-regulated DEPs.

C Heatmaps show the transcript and protein levels of the selected immunity-related genes in AMI samples along the AMI development. Each sample has three replicates.

D GSEA results: apoptosis pathway is activated during the development of AMI.

E Heatmaps show the transcript and protein levels of the selected apoptosis-related genes in AMI samples along the AMI development. Each sample has three replicates.

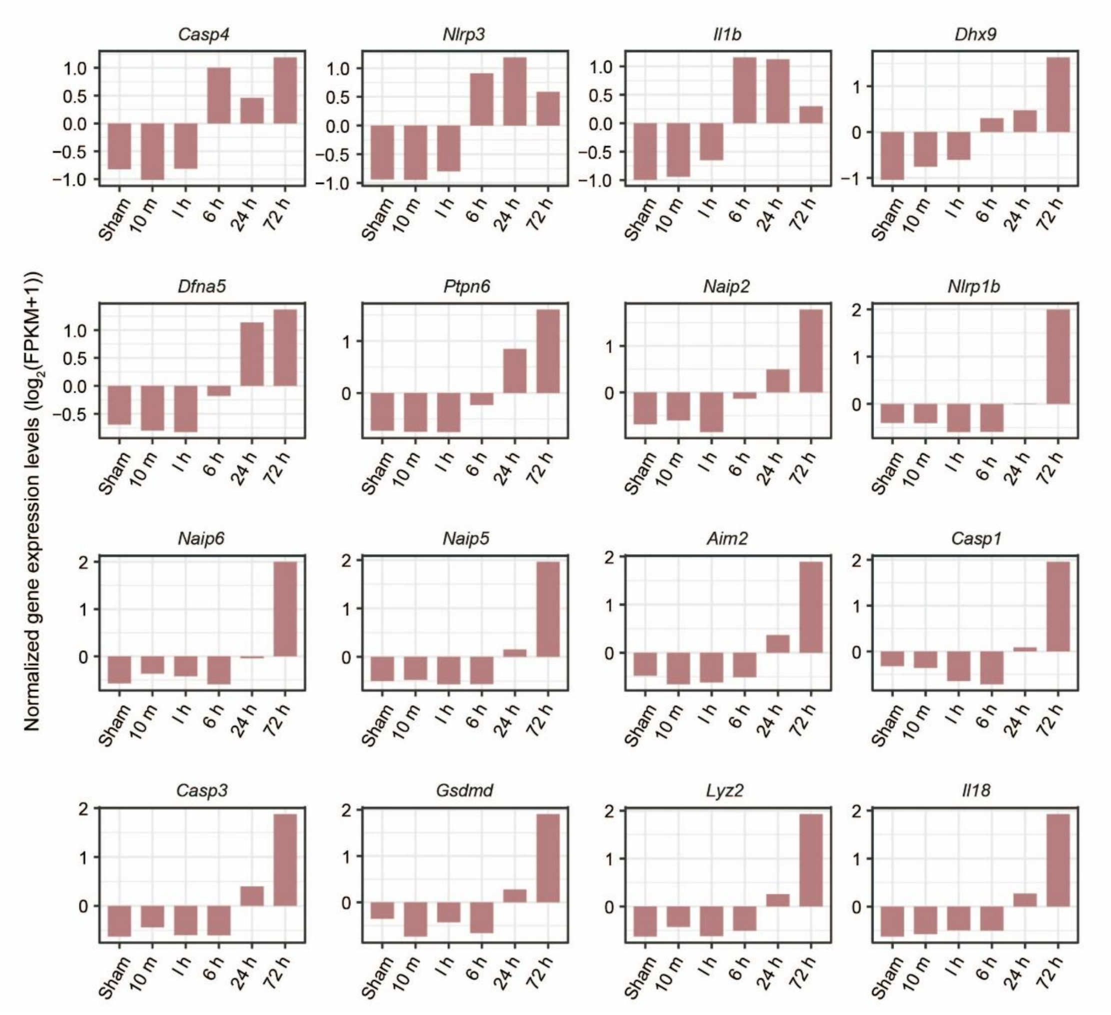

Supplementary Figure 3 The expression patterns of the genes from the pyroptosis interaction network (Fig. 3g) during the AMI development.

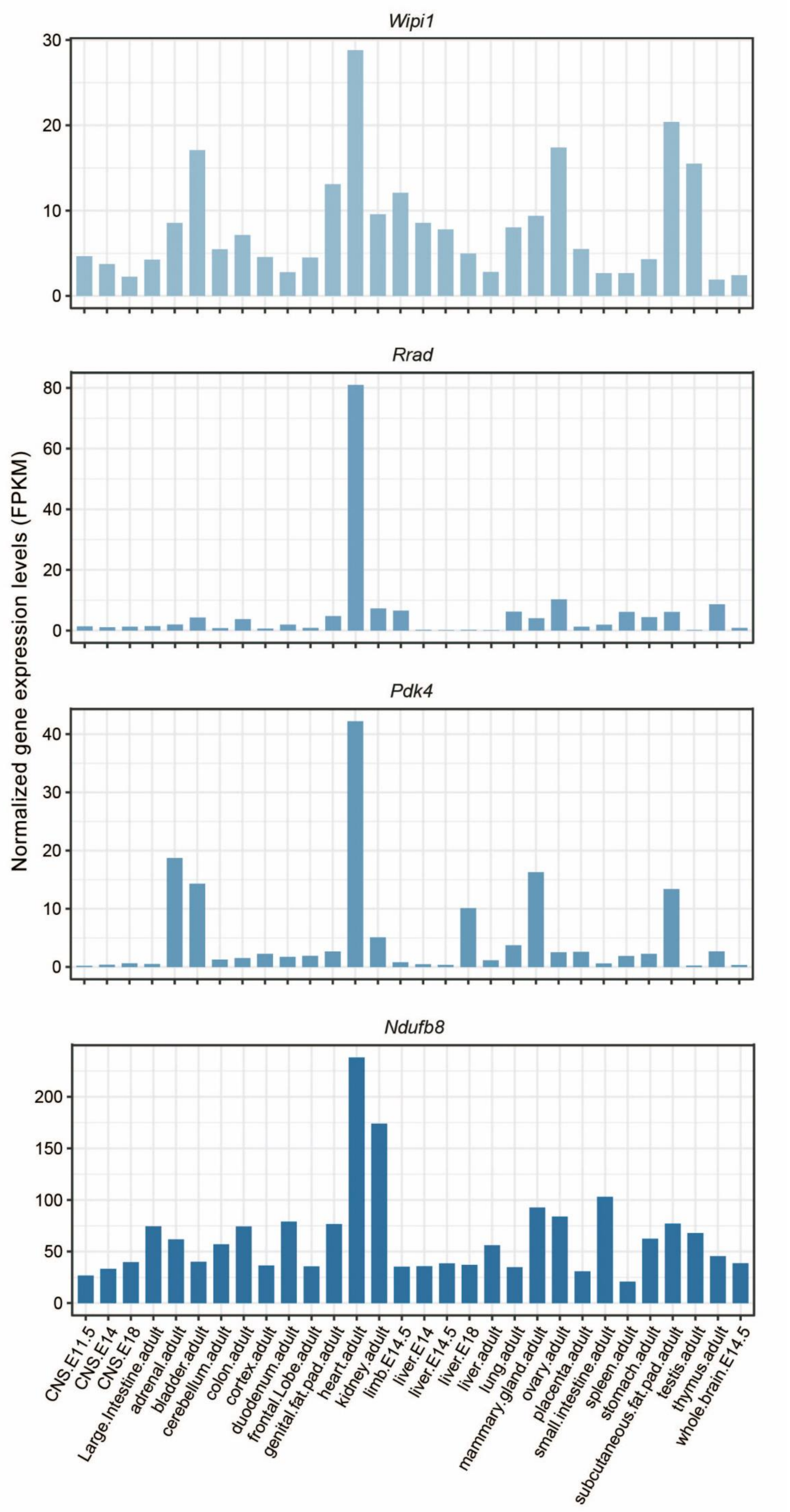

Wipi1
Rrad
Pdk4
Ndufb8
Normalized gene expression levels (FPKM)
0
10
20
30
40
60
80
50
100
150
200
CNS.E11.5
CNS.E14
CNS.E18
Large.Intestine.adult
adrenal.adult
bladder.adult
cerebellum.adult
colon.adult
cortex.adult
duodenum.adult
frontal.Lobe.adult
genital.fat.pad.adult
heart.adult
kidney.adult
limb.E14.5
liver.E14
liver.E14.5
liver.E18
liver.adult
lung.adult
mammary.gland.adult
ovary.adult
placenta.adult
small.intestine.adult
spleen.adult
stomach.adult
subcutaneous.fat.pad.adult
testis.adult
thymus.adult
whole.brain.E14.5

Supplementary Figure 4 Bar plots show the expression levels of *Wipi1, Rrad, Pdk4* and *Ndufb8* in different tissues. Data are from PRJEB4337.

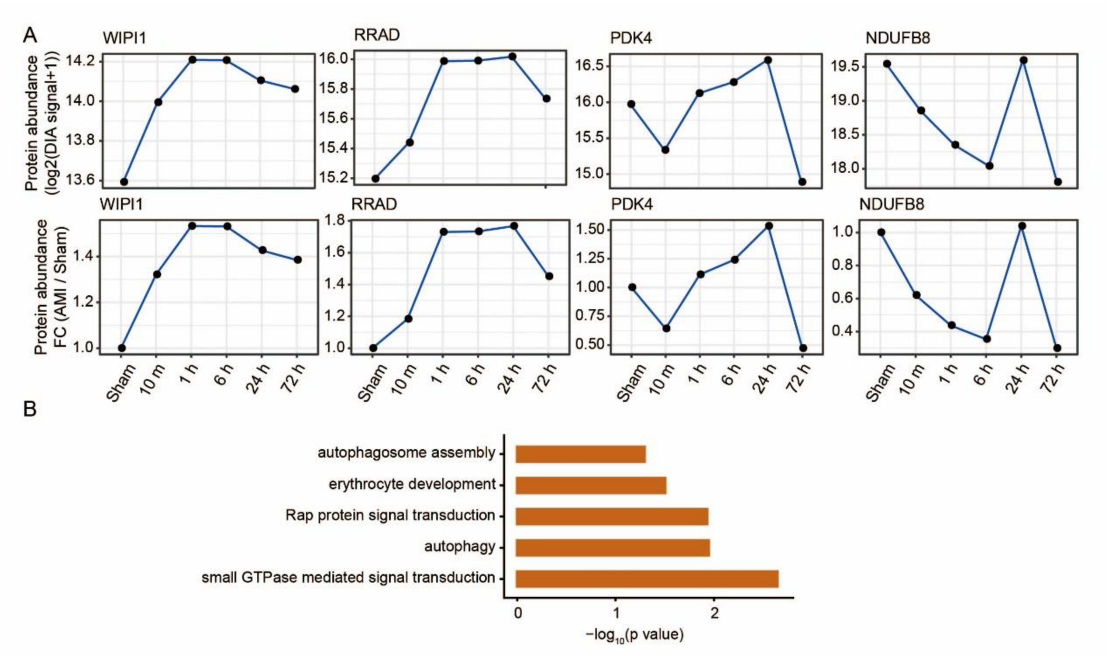

Supplementary Figure 5 The protein levels of the potential early markers for AMI.

A Line plots show the protein abundance levels of the potential early markers for AMI. Top: the absolute abundance. Bottom: the abundance normalized by the level of the sham samples.

B Barplot shows the significantly enriched GO terms (biological processes) of up-regulated DEPs between the 1 h AMI samples and the sham samples.

Supplementary Table 1 The qRT-PCR primers used in this study.

name	sequence
Gapdh-PF	5’ AGGTCGGTGTGAACGGATTTG 3’
Gapdh–PR	5’ TGTAGACCATGTAGTTGAGGTCA 3’
Gsdmd-PF	5’ CCATCGGCCTTTGAGAAAGTG 3’
Gsdmd-PR	5’ ACACATGAATAACGGGGTTTCC 3’
Casp1-PF	5’ ACAAGGCACGGGACCTATG 3’
Casp1-PR	5’ TCCCAGTCAGTCCTGGAAATG 3’
